# Supplementary material for: Physical and mental health professionals perspectives of providing mental health care for young people: A qualitative interview study
Source: PLOS Ment Health. 2026 Feb 9;3(2):e0000542. doi: 10.1371/journal.pmen.0000542 (PMC12885261; doi:10.1371/journal.pmen.0000542)
Supplement: S1 File — (DOCX) [file pmen.0000542.s001.docx]

S1 File: Anonymised Interview Guide

| 1 | In your current role, do you interact with staff from the other NHS trust? |
| --- | --- |
| 1a | What is the nature of those interactions? |
| 2 | What is your experience of those interactions? |
| 2a | What is positive? |
| 2b | What could be better? |
| 3 | How would you hope to interact/work together in the Children’s Hospital? |
