# Supplementary material for: Physical and mental health professionals perspectives of providing mental health care for young people: A qualitative interview study
Source: PLOS Ment Health. 2026 Feb 9;3(2):e0000542. doi: 10.1371/journal.pmen.0000542 (PMC12885261; doi:10.1371/journal.pmen.0000542)
Supplement: Attachment — Submitted filename: Response to Reviewers.docx [file pmen.0000542.s003.docx]

Reviewer's Responses to Questions

**Comments to the Author**

1. Does this manuscript meet PLOS Mental Health’s [publication criteria](https://journals.plos.org/mentalhealth/s/criteria-for-publication)? Is the manuscript technically sound, and do the data support the conclusions? The manuscript must describe methodologically and ethically rigorous research with conclusions that are appropriately drawn based on the data presented.

Reviewer #1: Partly

Reviewer #2: Yes

2. Has the statistical analysis been performed appropriately and rigorously?

Reviewer #1: N/A

Reviewer #2: N/A

3. Have the authors made all data underlying the findings in their manuscript fully available (please refer to the Data Availability Statement at the start of the manuscript PDF file)?

The [PLOS Data policy](https://journals.plos.org/mentalhealth/s/data-availability) requires authors to make all data underlying the findings described in their manuscript fully available without restriction, with rare exception. The data should be provided as part of the manuscript or its supporting information, or deposited to a public repository. For example, in addition to summary statistics, the data points behind means, medians and variance measures should be available. If there are restrictions on publicly sharing data—e.g. participant privacy or use of data from a third party—those must be specified.

Reviewer #1: No

Reviewer #2: Yes

4. Is the manuscript presented in an intelligible fashion and written in standard English?

PLOS Mental Health does not copyedit accepted manuscripts, so the language in submitted articles must be clear, correct, and unambiguous. Any typographical or grammatical errors should be corrected at revision, so please note any specific errors here.

Reviewer #1: Yes

Reviewer #2: Yes

5. Review Comments to the Author

Please use the space provided to explain your answers to the questions above. You may also include additional comments for the author, including concerns about dual publication, research ethics, or publication ethics. (Please upload your review as an attachment if it exceeds 20,000 characters)

|  | Comments | Author’s response |
| --- | --- | --- |
| Reviewer 1 | Sample is small (15 only). The authors should say this clearly and why it is enough for their question. | Thank you for this reflection. We have added insights from the additional interviews done with mental health professionals to our manuscript, bringing the total sample up to 31 participants. We have also added a clarifying statement of how this sample size is considered sufficient by others in the field to reach information power and saturation. We have also updated our methods and research question to better align with the new combined population. |
| Reviewer 1 | Participants look too similar (mostly one gender/ narrow roles ). The authors add more variety, or admit this limit properly and explore how this can bias their conclusion | Thank you for this reflection. We have added a point about how all our participants were women to our limitations section and considered how this may bias the conclusions made. |
| Reviewer 1 | They used group and one-to-one interviews together; explain what each format added and if answers differ or not.  And how they handled themes or response that did not match the majority view | We have added detail in the data analysis sub-section under the methods section (Line 198 - 206) about how participants chose to have either one-to-one or group interviews, as participants chose the interview setting due to scheduling conveniences .  We included responses that did not match the majority view. We have made it clearer in our results section when quotes are from divergent/negative cases that differed from the majority of our interviews. |
| Reviewer 1 | Some interviews are short; the authors justify how enough depth still came, with typical time given | We have added detail to our methods section (Line 212 - 219) of the strategies we implemented to ensure we are confident that enough depth came from the interviews that were short due to pragmatic limitations of having to conduct interviews during staff lunch breaks. |
| Reviewer 1 | Ethics and consent for re-use must be stated plainly; confirm that approval cover this secondary analysis. | We have included the following clause in our ethics statement on line 141 “including permission to conduct this secondary analysis” |
| Reviewer 1 | The authors tell who did interviews/coding, their background, and how bias was reduce. | We have added identifying details, relevant demographic characteristics and initials throughout the methods section to clearly identify which authors supported which part of the data collection and analysis.  We have added details about how we used triangulation to help reduce bias in our interpretation (Line 240 – 258). |
| Reviewer 1 | Make the analysis steps very clear in 3–4 simple step; add a tiny theme map also if possible | We have created Figure 1 to present our analysis steps in a simple format and Figure 2 to add a theme map to our results section. |
| Reviewer 1 | Use person-first, simple words and define local terms (like “specialing”) for outside readers. | We have added relevant definitions for local medical terms |
| Reviewer 1 | Do not over-generalise; keep claims to these two trusts only. | Thank you for this comment. We have made edits to ensure our manuscript does not over-generalise |
| Reviewer 1 | Share de-identified material (interview guide, codebook, quote bank) and say how transcript can be access with request | Due to stipulations from the funder and ethics board we are unable to share transcripts on request.  The full interview guide has been uploaded alongside the requirements for resubmission as a supplementary file.  The codebook has been included in the manuscript, in the form of the theme map seen in Figure 2.  An anonymised quote bank, including the additional quotes we have added as part of this resubmission, can be accessed by email request to the corresponding author. |
| Reviewer 1 | Clean up title, abstract and references; keep consistent British spelling. | We have ensured that British spelling is consistent throughout the manuscript |
| Reviewer 2 | This is a thoughtful, well-written study of physical health professionals’ (clinicians et al.) perspectives on their experiences taking care of young persons’ mental health (MH) needs in non-MH settings – emergency rooms and pediatric inpatient units. The three main themes emerging from interviews of 15 physical health professionals (3 pediatricians, 5 nurses and health care assistants, 2 managers, 5 allied health care professionals – all women) were feeling out of their depth, their facilities’ being a MH waiting room, and their professional responsibility to keep their MH patients safe in non-MH settings. These themes ring true; they are equally applicable to the circumstances in our county hospital in the United States, where extended ER and general pediatric unit stays are used to compensate for severe lack of pediatric MH facilities. That this study was commissioned in advance of the building of a new Children’s Hospital and, one hopes, will be used to inform the design of the hospital (especially adequacy of MH spaces in the ER and main wards), is commendable. | Thank you for your deep engagement with the paper. |
| Reviewer 2 | All this being said, however, I feel I have been served only half a loaf – where are the corresponding interview analyses and quotes from the 16 MH professionals also interviewed? Given the clear, informative exposition provided thus far for the 15 physical health professionals, I believe similar data about the 16 MH professionals are essential to complement the current presentation. It is likely these 16 MH professionals had comments about the physical health professionals’ being highly anxious about certain MH patients, demanding the MH clinicians do something right away, complaining of the long delays between their being consulted and their appearance at the bedside, etc. (This from our own hospital experiences.). A telling point is that the physical health clinicians believed they had to correct their patients’ physical issues before a MH clinician would provide a consult (lines 314-316). So, I strongly urge the authors to complete this report by providing parallel information on the attitudes and main themes of the 16 MH professionals. This will provide a considerably more powerful and influential report, especially locally for use in the new hospital design, as well as around the world (e.g., in our advocating for increased MH services for young people in our area of the U.S.).  Lines 408-410: A tease; as I strongly urged above, please also present the MH professionals’ findings in this report. | Thank you for this reflection. We have added insights from the mental health professionals interviews to our manuscript. We have also updated our methods and research question to better align with the new population |
| Reviewer 2 | Title: …professionals’…. (plural) Line 84: Physical healthcare professionals are increasingly… Line 118: …which they are struggling to find… Who are “they”? Could end sentence with …mental health needs. Line 136: …data were shared. (Plural.) Line 159: …was reflected (Table 1). (Diversity is singular. Add Table 1 here and move up Table after this paragraph.)  Line 193: …were complete, | Corrections to grammatical inconsistencies that you identified have been made |
| Reviewer 2 | Table 1: All participants were women. Possibility of sex bias; e.g., heightened concern about ensuring the safety of aggressive male adolescents and their own safety? | Thank you for this reflection. We have added this point about how the participants were all women to our limitations section and considered how it may bias the conclusions made |
| Reviewer 2 | Lines 421-424: This isn’t a limitation; it’s one of the purposes of the report | Thank you for this reflection. We have made it clearer that the snapshot account the paper offers is the purpose of the report rather than a limitation. |
| Reviewer 2 | References: Please be consistent in the citations; some article titles are lower case, others are in caps. Please space after all the initial numbers (e.g., ref’s 10-13). If DOIs are available, it’s a good idea to include them for ease of searching. | References have been reformatted and DOIs where available have been added |
